# A comparative study on intraocular pressure under various anesthetics in cynomolgus monkeys (*Macaca fascicularis*)

**DOI:** 10.1186/s42826-021-00092-2

**Published:** 2021-06-22

**Authors:** Hong-Soo Lee, Da-Hee Kim, Sung-Hwan Kim, Min-Sung Kang, Han Na Suh

**Affiliations:** 1 Biotoxtech, 53 Yeongudanji-ro, Ochang-eup Chungcheongbuk-do, South Korea; 2 Korea Institute of Toxicology, 30 Haekhak 1-gil, Jeongeup Jeollabuk-do, South Korea

**Keywords:** Anesthetics, Cynomolgus monkey, Intraocular pressure, TonoVet™

## Abstract

**Background:**

Nonhuman primates (NHPs) are superior model for ocular research due to its morphological and physiological similarities with humans. Thus, the effect of four different anesthetic combinations [ketamine (10 mg/kg), ketamine + xylazine (7 + 0.6 mg/kg), zoletil (4 mg/kg), and zoletil + xylazine (4 + 0.2 mg/kg)] on intraocular pressure (IOP) was determined in cynomolgus monkeys.

**Results:**

The administration of ketamine + xylazine or zoletil + xylazine resulted in lower IOP compared to ketamine or zoletil alone. Moreover, the IOP in male monkeys was higher than in females. The difference between the right and left eye was not found.

**Conclusions:**

Anesthetics affected the IOP, and gender differences should be considered when measuring the IOP of nonhuman primates (NHPs).

## Background

Preclinical study is a step to examine the drug feasibility and collect the drug safety data prior to clinical trial. Generally, regulatory guidelines indicate the use of rodent (mouse and rat) and/or non-rodent [nonhuman primates (NHP), porcine, canine] in preclinical study. To select the right species, pharmacological relevance based on literature, *in silico*, *in vitro*, *ex vivo*, and *in vivo* data should be considered [1, 2]. The canine is a proper testing species for new chemical entities. On the other hand, biotherapeutics such as monoclonal antibody, antisense oligonucleotide, or recombinant protein have potent pharmacological activity in NHPs [3, 4]. Porcine is the best model for dermal toxicity due to the histological and functional similarity of its skin to humans. Thus, minimum and proper NHP application is required in preclinical safety or efficacy study.

The intraocular pressure (IOP) is the pressure within the eye, which is based on the production and drainage of aqueous humor. IOP can be affected by various factors such as obesity [5], water consumption [6], stress [7], circadian rhythm [8], reproductive cycle [9], systemic blood pressure [10], and anesthesia [11]. In addition, ocular toxicity may be associated with IOP changes during the preclinical study. NHPs are superior model for ocular research due to its morphological and physiological similarities with humans [9, 12]. All drugs which are clinical prescribed against glaucoma have been found to reduce the IOP in NHPs. The development of novel glaucoma medication is widely ongoing, utilizing NHPs [13, 14]. Thus, an accurate, reproducible, and convenient IOP assessment for NHP is essential in preclinical studies.

To acquire the accurate IOP of NHPs, IOP is measured under anesthesia. Ketamine, a NMDA receptor antagonist, is commonly used as an anesthetic agents in NHPs [15]. Combination of ketamine with other dissociative anesthetics attenuates the reflex during anesthesia [16]. Zoletil is a veterinary anesthetic combination of tiletamine hydrochloride and zolazepam hydrochloride. Tiletamine is a NMDA receptor antagonist and zolazepam is an enhancer of GABA action having sedative, anxiolytic, and skeletal muscle relaxant effect [17]. Xylazine, an adrenergic receptor α2 agonist, is used for sedation, anesthesia, muscle relaxation, and analgesia in animals. Here we have investigated the effect of different combinations of anesthetics on the IOP of NHPs using TonoVet™. This study may serve as a foundation of the IOP baseline when measuring the IOP in NHPs.

## Results

### Effect of four different combination of anesthetics on the IOP of cynomolgus monkeys

**Fig. 1:**
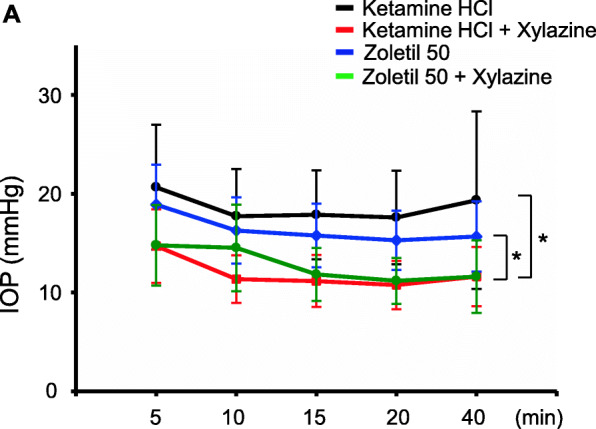
Effect of anesthetics on the IOP in cynomolgus monkeys. (**A**) IOP was measured (5, 10, 15, 20, and 40 min) after anesthetization. Xylazine-treated animals showed significantly lower IOP compared to ketamine or zoletil alone-treated animals. *p < 0.05. n = 20 in each group (both eyes of five male and five female monkeys)

To determine the effect of anesthetics on IOP, we chose four different anesthetic combinations; ketamine, ketamine + xylazine, zoletil, or zoletil + xylazine. During the IOP measurement in NHPs, ketamine was used for sedation [18]. The animals under ketamine showed the steady IOP (20.65 ± 6.34 to 19.35 ± 9.00 mmHg), supporting ketamine as the suitable anesthetic during IOP measurement. However, ketamine + xylazine showed decreased IOP (14.7 ± 3.73 to 11.6 ± 3.00 mmHg). When we compared the IOP at 5 min, ketamine + xylazine showed the lower IOP than ketamine alone. Zoletil is a more potent anesthetic regulating both NMDA receptor and GABA. We found that zoletil slightly decreased the IOP (18.9 ± 4.06 to 15.68 ± 3.57 mmHg), and zoletil + xylazine had similar decreased pattern (14.78 ± 4.08 to 11.6 ± 3.67 mmHg) (Fig. 1). Above all, ketamine had steady IOP, and adding xylazine decreased the IOP.

### Comparison of IOP between two genders and right/left eyes in cynomolgus monkeys

**Fig. 2:**
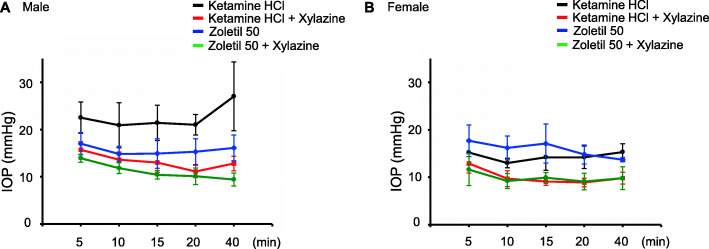
A comparative study of IOP in male and female cynomolgus monkeys. (**A-B**) IOP was measured (5, 10, 15, 20, and 40 min) after anesthetization. The IOP in male (**A**) was higher than in female (**B**). *p < 0.05. *n* = 10 in each group (both eyes of five male or five female monkeys)

To determine whether the IOP is affected by gender, we compared the IOP of female and male monkeys. We found that the male had higher IOP (from 22.5 ± 3.30 to 27 ± 7.29 mmHg) than the female (from 15.2 ± 2.64 to 15.3 ± 1.75 mmHg) in ketamine. Other anesthetics were not different between genders (Fig. 2).

**Fig. 3:**
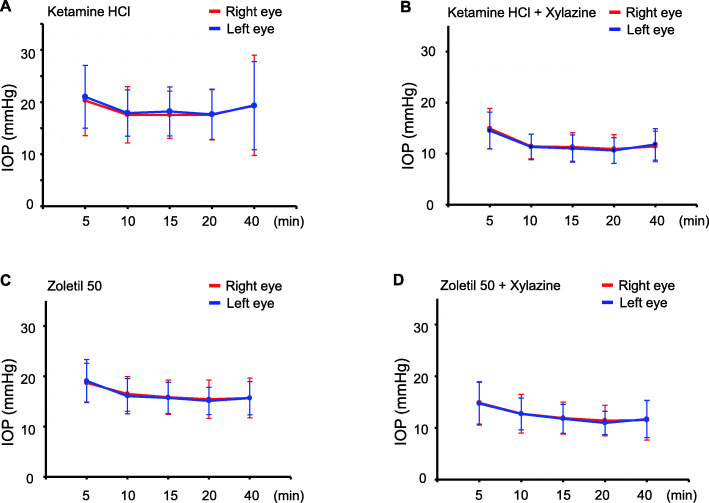
A comparative study of IOP in right and left eyes. (**A-D**) IOP was measured (5, 10, 15, 20, and 40 min) after anesthetization. There was no difference in the IOP of right and left eyes. *p < 0.05. *n* = 10 in each group (right or left eyes in both genders)

To examine whether the IOP had variations in both eyes, we analyzed the IOP of the right and left eye. The right and left eye had similar IOP values regardless of the anesthetic types (Fig. 3).

## Discussion

In this study, we examined the IOP to compare the effects of four different anesthetics in male and female Vietnamese cynomolgus monkeys using rebound tonometer, TonoVet™. As cynomolgus monkeys can be a representative ocular disease model and are highly suitable for drug screening for glaucoma [12], we have utilized this strain for IOP measurement. TonoVet™ was validated as an accurate equipment for IOP measurement due to the high correlation with the actual eye pressure [18]. Another study found that the IOP values have greater variety when using TonoVet™ compared to fixed tonometer [19]. Thus, technical consistency and proper position of TonoVet™ are essential to get reproducible IOP.

To acquire the representative IOP, NHPs were under general anesthesia during the procedure. It was reported that ketamine increases the IOP in cats, dogs, and rabbits. Ketamine with diazepam significantly elevates the IOP in dogs [13]. On the other hand, midazolam decreases the IOP in humans [20]. The median IOP was measured to be 10 to 25 mmHg in most animals [21, 22]. We found that all measured IOP was in normal range, regardless of anesthetics used. The α2-adrenoceptor agonist, xylazine, decreased the IOP by reducing aqueous flow [23]. Of note, topical or systemic administration of atropine sulfate increases canine IOP [24]. As atropine is an anticholinergic drug, the differential effect of atropine on combined anesthetics is possible. Lions anesthetized with ketamine showed similar IOP regardless of atropine [25]. The possible interference or synergistic effect of combination of various anesthetics is need to be explored. As anesthetics affect the IOP, it should be noted that the basal IOP depends on the anesthetics when a researcher performs an ophthalmologic procedure.

Gender is another factor associated with the IOP in humans and lions [25, 26]. We found that ketamine-treated male had higher IOP than female monkeys. Other anesthetic did not show the difference in IOP between the two genders. The differences of IOP based on gender might be related to sexual hormone or anesthetic sensitivity. Here, we demonstrated that xylazine decreased the IOP significantly after administration, regardless of ketamine or zoletil combination. This study provides the baseline IOP of cynomolgus monkeys under various anesthetics, which might be helpful for scientists and pharmaceutical companies interested in glaucoma and eyes.

## Conclusions

Anesthetics affected the IOP, and gender differences should be considered when measuring the IOP of nonhuman primates (NHPs).

## Methods

### Animals, Husbandry, and Feeding

Ten cynomolgus monkeys, (five males and five females; *Macaca fascicularis*), aged 2–3 year and weight 2.89–3.17 kg, supplied from Nafo Vanny (Dong Nai, Vietnam). Monkeys were housed individually in a stainless cage (540mm x 760mm x 850mm) with enrichments. Room temperature and humidity regulated (20 ~ 29 °C; 30 ~ 70 %). Fluorescent lighting 300–700 lx and air changes 10–20 times/hour maintained. Water was provided *ad libitum* and food was provided at the rate of 3 % of the body weight per day. Fruits were provided twice a week. All the animal experiments were conducted under Institutional Animal Care and Use Committee guideline of the Korea Institute of Toxicology (IACUC KIT 1506 − 0158).

### Intraocular pressure measurements

Any ophthalmic abnormalities were not detected during this procedure, as confirmed by slit-lamp biomicroscopy and funduscopy. The animals were pre-medicated with atropine sulfate (0.04 mg/kg, SC), followed by ketamine (10 mg/kg, IM), ketamine + xylazine (7 mg/kg + 0.6 mg/kg, IM), zoletil (4 mg/kg, IM), or zoletil + xylazine (4 mg/kg + 0.2 mg/kg, IM). Then, IOP was measured in each eyes at 5, 10, 15, 20, and 40 min after anesthetization. IOP was measured bilaterally using the rebound tonometer (TonoVet™, Vantaa, Finland), following the manufacturer’s protocol. The right eye was measured first. TonoVet™ software was programmed for six measurements (consecutive central corneal touches) and displayed the average of six values. All measurements were conducted in the same room between 6 pm and 8 pm to minimize circadian variation.

### Statistical analysis

The data were analyzed using the statistical package SPSS (SPSS Inc., IL, USA) for Windows version 12.0. Two-way ANOVA with repeated measures were used, followed by Bonferroni’s test to compare the average IOP values of both eyes among groups. For IOP comparison between the right and left eye, Student’s T-test was used. Levene’s test was applied for the homogeneity of variance in both genders. The data are presented as the mean ± standard deviation. The differences are considered significant when *p* < 0.05.

## Data Availability

All data are included in Results.

## Abbreviations

NHP: Nonhuman primates
IOP: Intraocular pressure
